# Editorial: Investigating the integration of family functioning and perinatal psychiatry

**DOI:** 10.3389/fpsyt.2026.1940773

**Published:** 2026-09-10

**Authors:** Mohammadreza Shalbafan, Yao Cheng, Jieyun Yin

**Affiliations:** 1 Mental Health Research Center, Psychosocial Health Research Institute, Department of Psychiatry, School of Medicine, Iran University of Medical Sciences, Tehran, Iran; 2 Obstetrics Department, Maternal and Child Health Hospital of Hubei Province, Wuhan, China; 3 Soochow University, Suzhou, China

**Keywords:** family relations, maternal health, mental disorders, mental health, perinatal care, postpartum period, pregnancy, social support

Perinatal psychiatry has become one of the most important fields in psychiatry due to growing healthcare and public awareness of the importance of the mental health of pregnant and postpartum women (1). This increasing attention is reflected not only in the expanding body of scientific research but also in the prioritization of perinatal mental health within public mental health systems as a key component of routine clinical care and mental and psychosocial support in crisis- and conflict-affected settings (2–4). Within this context, the family, particularly in traditional and transitional cultures, represents one of the most influential social systems and can serve as either a major source of support or a substantial source of stress for both parents and their children. Given the importance of these topics and the relatively limited research integrating family functioning with perinatal psychiatry, this Research Topic, ‘Investigating the Integration of Family Functioning and Perinatal Psychiatry’, brings together seven articles from diverse countries and research settings addressing different aspects of this relationship.

Pang et al. investigated the association between family functioning and loneliness among 507 women undergoing home-based postpartum confinement who attended their 6-week postpartum follow-up visit at a maternal and child health hospital in Guangzhou, China. They reported a loneliness prevalence of 27.4%. Better family functioning was associated with lower levels of loneliness. Higher Lubben Social Network Scale-6 (LSNS-6) scores, reflecting greater social connectedness and a lower risk of social isolation, were also associated with lower loneliness and better family functioning. In addition, women without a stable postpartum income and primiparous women reported significantly higher loneliness scores.

Alharbi et al. investigated the effects of Kegel exercises on postpartum pain, pelvic floor dysfunction, and sexual function among women attending the Department of Obstetrics and Gynecology at the National Guard Hospital, King Abdul-Aziz Medical City, Jeddah, Saudi Arabia. Using a quasi-experimental design with pre- and post-intervention assessments conducted six weeks apart and 31 participants in each group, they found that Kegel exercises improved sexual function and reduced pain among women with postpartum complications.

Furthermore, Dai et al. presented a Hypothesis and Theory article proposing the family-dominant hypothesis, which emphasizes the influence of the family of origin on the mental health of offspring. The authors comprehensively discussed the theoretical framework, supporting evidence, potential mechanisms, and implications of this hypothesis.

Christnacht et al. reported a case series of three patients with postpartum mood and anxiety disorders in Seattle, Washington, who received ketamine-assisted psychotherapy. Across the cases, participants experienced improvements in mental health symptoms, psychosocial self-awareness, self-worth, and self-efficacy during the postpartum period.

Li et al. conducted a mixed-method study to develop and validate an instrument for assessing the personality masks of mothers with young children among 486 Chinese mothers. They concluded that the personality mask represents a valuable construct for understanding maternal behavior and that promoting balanced psychological adaptation may enhance parenting effectiveness.

Kannikeswaran et al. conducted another mixed-method study to evaluate healthcare providers’ perceptions of integrating Infant Mental Health Behavioral Health Consultants (IMH-BHCs) into obstetric clinics in Michigan, USA. The study explored the perceived effects on patient and provider outcomes, barriers to behavioral health integration, and the impact of incorporating behavioral health services into perinatal care. The authors noted that a substantial proportion of patients served by these clinics were Black, Indigenous, and People of Color (BIPOC). Quantitative findings demonstrated high levels of provider knowledge of and engagement with the model, while respondents reported improvements in clinical workflow, scope of care, and time management. Qualitative interviews identified two overarching themes with five subthemes: (1) initiation and engagement with the IMH-BHC model and (2) perceived effectiveness of the IMH-BHC. Overall, the integration of IMH-BHCs was considered beneficial in reducing provider workload, improving access to mental healthcare for socially marginalized populations, and enhancing patient engagement.

Finally, Ogawa et al. investigated risk and protective factors for child maltreatment among pregnant women with psychosocial difficulties by analyzing the electronic medical records of 253 consecutive women who received care and delivered at Chiba University Hospital, Japan. Maternal grandmother’s support and support from other family members emerged as protective factors, whereas maternal mental illness was identified as a risk factor. In addition, increasing maternal age was associated with a lower incidence of child maltreatment, suggesting adolescent pregnancy as another important risk factor.

Collectively, the studies included in this Research Topic highlight the bidirectional relationship between family functioning and perinatal mental health across diverse cultural and healthcare settings. They also underscore the need for further large-scale studies to strengthen the evidence base and inform future clinical practice and public health policy.
